# Supplementary material for: Carbamazepine Restores Neuronal Signaling, Protein Synthesis, and Cognitive Function in a Mouse Model of Fragile X Syndrome
Source: Int J Mol Sci. 2020 Dec 7;21(23):9327. doi: 10.3390/ijms21239327 (PMC7731004; doi:10.3390/ijms21239327)
Supplement: Supplementary file 1 [file ijms-21-09327-s001.pdf]

Supplementary Fig. 1, Ding et al.

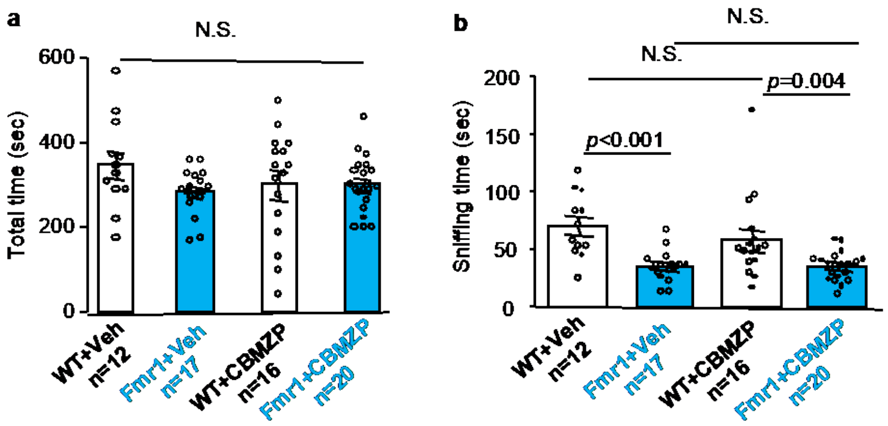

Supplementary Fig. 1 Single carbamazepine administration has no effects on social interaction. 2.5- to 3-month old male wild type (WT) and *Fmr1* KO mice were injected with vehicle (Veh) (10% DMSO) or 20 mg/kg carbamazepine (CBMZP). 60 min after the injection, mice were subjected to a 3-chamber social interaction test. Total time spent in the stimulus mouse chamber and direct social interaction are presented in **a** and **b**, respectively. Data are presented as average +/- SEM. **a.** there is no difference in total time among all groups (genotype effect: $F_{1, 61}$=1.944, p=0.168; treatment effect: $F_{1, 61}$=0.481, p=0.491; genotype X treatment interaction: $F_{1, 61}$=1.899, p=0.173). **b.** *Fmr1* KO mice show social deficits and are not affected by carbamazepine (genotype effect: $F_{1, 61}$=24.088, p<0.001; treatment effect: $F_{1, 61}$=0.864, p=0.361; genotype X treatment interaction: $F_{1, 61}$=0.946, p=0.335). Difference between two groups was determined by *post hoc* analysis following two-way ANOVA test. N.S.: not significant.

Supplementary Fig. 2, Ding et al.

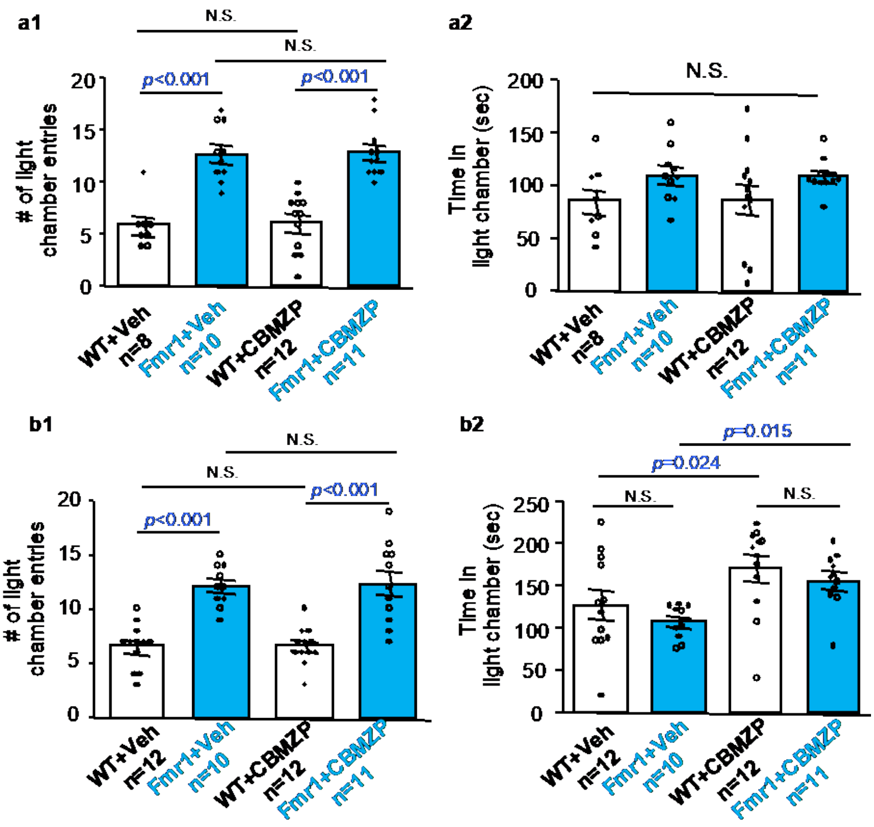

Supplementary Fig. 2 Carbamazepine does not have effect on the abnormal light-dark test behavior in *Fmr1* KO mice. 2.5- to 3-month old male wild type (WT) and *Fmr1* KO mice were i.p. injected with vehicle (Veh) (10% DMSO) or 20 mg/kg carbamazepine (CBMZP) once (**a**) or daily for 8 days (**b**). 60 min after the single injection (**a**) or the last daily injection (**b**), mice were subjected to a 5-min light dark test, during which animals were allowed to travel freely between the light and dark chamber. Number of transitions made between the light and dark chamber was recorded and presented in **a1** (genotype effect: $F_{1,37}$=68.412, p<0.001; drug effect: $F_{1,37}$=0.059, p=0.81; genotype X drug interaction: $F_{1,37}$=0.012, p=0.913) and **b1** (genotype effect: $F_{1,41}$=57.775, p<0.001; drug effect: $F_{1,41}$=0.031, p=0.860; genotype X drug interaction: $F_{1,41}$=0.031, p=0.860). Total time spent in the light chamber is presented in **a2** (genotype effect: $F_{1,37}$=4.510, p=0.04; drug effect: $F_{1,37}$=0.005, p=0.944; genotype X drug interaction: $F_{1,37}$=0.020, p=0.888) and **b2** (genotype effect: $F_{1,41}$=1.677, p=0.230; drug effect: $F_{1,41}$=11.874, p=0.001; genotype X drug interaction: $F_{1,41}$=0.063, p=0.804). Data are presented as average +/- SEM. Difference between two groups was determined by *post hoc* analysis following two-way ANOVA test. N.S.: not significant.

Supplementary Fig. 3, Ding et al.

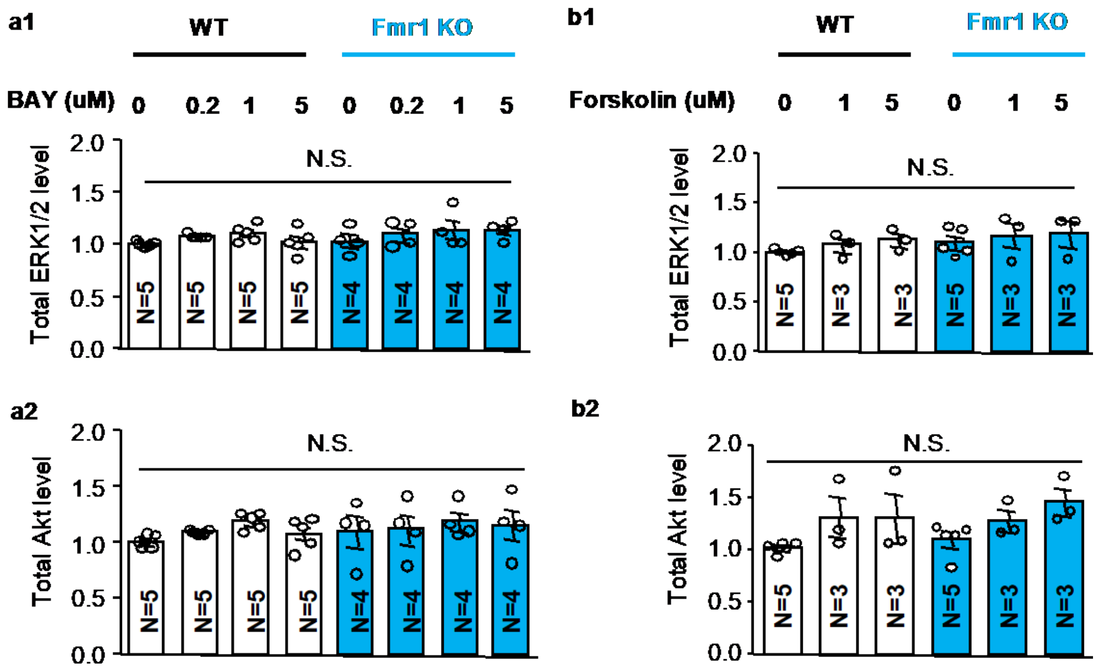

Supplementary Fig. 3 Effects of BAY607550 (**a**) and forskolin (**b**) on the level of total ERK1/2 and Akt in wild type and *Fmr1* KO neurons. DIV 14 neurons were treated with BAY607550 and forskolin as described in Fig. 5. Quantification of ERK/12 (**a1** and **b1**) and Akt (**a2**, and **b2**) is presented as average +/- SEM following normalization with β-actin. The N number indicated in the figure is the number of experiments performed with independent neuronal cultures. The p values were determined by two-way ANOVA. N.S.: not significant. **a1**. genotype effect: $F_{1,28}$=1.639, p=0.211; drug effect: $F_{3,28}$=1.535, p=0.227; genotype X drug interaction: $F_{3,28}$=0.322, p=0.809. **a2**. genotype effect: $F_{1,28}$=0.630, p=0.434; drug effect: $F_{3,28}$=0.963, p=0.424; genotype X drug interaction: $F_{3,28}$=0.143, p=0.933. **b1**. genotype effect: $F_{1,16}$=1.949, p=0.182; drug effect: $F_{2,16}$=1.097, p=0.358; genotype X drug interaction: $F_{2,16}$=0.024, p=0.976. **b2.** genotype effect: $F_{1,16}$=0.473, p=0.501; drug effect: $F_{2,16}$=4.320, p=0.032; genotype X drug interaction: $F_{2,16}$=0.317, p=0.733.
